# P-1475. Immunogenicity of two vaccinations of an H5N8 MF59-Adjuvanted Cell Culture-derived Influenza Vaccine (aH5N8c) in Healthy Subjects ≥18 Years of Age

**DOI:** 10.1093/ofid/ofaf695.1661

**Published:** 2026-01-11

**Authors:** Janine Oberije, Adam Brosz, Eve Versage, Esther Van Twuijver, Matthew Hohenboken

**Affiliations:** CSL Seqirus, Amsterdam, Noord-Holland, Netherlands; Velocity Research, Grand Island, Nebraska; CSL Seqirus USA, Waltham, Massachusetts; CSL Seqirus, Amsterdam, Noord-Holland, Netherlands; CSL Seqirus, Amsterdam, Noord-Holland, Netherlands

## Abstract

**Background:**

Pathogenic avian influenza is considered a global threat. This Phase 2 study assessed the immunogenicity of two doses of MF59-adjuvanted cell culture-derived H5 influenza vaccine administered three weeks apart in adults aged ≥18 years. Primary endpoints included hemagglutination inhibition (HI) and microneutralization (MN) antibody responses against H5N8 at Day 43. Safety results are reported separately.

**Figure:**
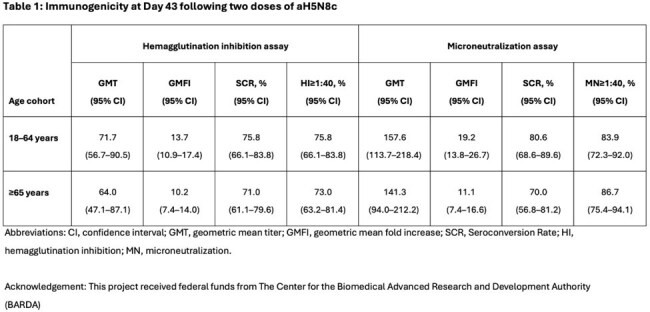

**Methods:**

The study enrolled 479 subjects of whom 239 subjects received two doses of aH5N8c. Participants included 121 aged 18–64 years, of which 60 were poultry workers, and 118 aged ≥65 years. Serum samples were collected at baseline (Day 1), Day 22, and Day 43 to assess HI and MN antibody responses. Analyses included geometric mean titers (GMTs), geometric mean fold increases (GMFIs), seroconversion rates (SCRs), and percentages achieving titers ≥1:40. Subgroup analyses were conducted by age, poultry worker status, prior influenza vaccination, sex, and race.

**Results:**

Following the first dose of aH5N8c, an increase in HI titers was observed, with GMFIs of 3.8 in the 18–64 years cohort and 5.6 in the ≥65 years cohort at Day 22. Following the second dose, HI titers continued to rise, reaching GMFIs of 13.7 in younger and 10.2 in older adults at Day 43. SCRs were 75.8% in younger adults and 71.0% in older adults; percentages achieving HI titers ≥1:40 were 75.8% and 73.0%, respectively. *Table 1.*MN titers as assessed in a subset were generally higher than HI titers, showing robust antibody responses across age cohorts at Day 43. MN GMTs exceeded HI GMTs, with Day 43 MN GMTs of 157.6 in younger adults and 141.3 in older adults. MN SCRs reached 80.6% in younger and 70.0% in older adults. *Table 1.* No notable differences in immune responses by age, poultry worker status, prior influenza vaccination, sex, or race were observed; baseline titers were uniformly low across subgroups.

**Conclusion:**

Two doses of aH5N8c three weeks apart elicited strong H5N8-specific antibody responses by Day 43 as measured by HI and MN assays. Responses were consistent across age cohorts and subgroups, including poultry workers and those with prior influenza vaccination history. These data further support MF59-adjuvanted cell-based monovalent influenza vaccines as an important, immunogenic public health tool against H5 influenza.

**Disclosures:**

Janine Oberije, PhD, CSL Seqirus: Employee Adam Brosz, MD, CSL Seqirus: Advisor/Consultant Eve Versage, MA, CSL Seqirus: Employee Esther Van Twuijver, PhD, CSL Seqirus: Employee Matthew Hohenboken, MD, PhD, CSL Seqirus: Employee

